# Supplementary material for: Seismic resolution improving by a sequential convolutional neural network
Source: PLoS One. 2024 Jun 11;19(6):e0304981. doi: 10.1371/journal.pone.0304981 (PMC11166346; doi:10.1371/journal.pone.0304981)
Supplement: Attachment — Submitted filename: Response to Reviewers -v2.docx [file pone.0304981.s008.docx]

**Comment 1:** What is the difference or contribution of the authors as compared to the conference paper, Yuan et al. 2021. Improving Seismic Resolution by a Sequential Convolutional Neural Network. 82nd EAGE Annual Conference & Exhibition
**Response 1:** The conference paper is an effectiveness inquiry of sequential convolutional neural network (SCNN) model applied in seismic resolution processing, this manuscript is a development of the cited conference paper, and there are some big differences. It is known that the quality and size of the training dataset are crucial to the effectiveness of deep learning models. In the cited conference paper, input and output dataset were generated from 14 well loggings by Ricker wavelet and wide-band Ricker wavelet. However, in this manuscript, there different wavelets were analyzed in section “3.1 Wavelet selection”, the wide-band B-spline wavelet exhibited the best ability for object distinction and was chosen to generate high-resolution (HR) seismic traces. In addition to the 14 wells, the Marmousi2 model containing more than 13,000 traces was adopted to generate low-resolution and high-resolution seismic traces, providing a large dataset for HR seismic processing deep learning. Base on the new training dataset, the HR processing SCNN model achieved an improved training and evaluation performance. Furthermore, the trained model was applied to both raw poststack and prestack seismic data from one practical survey, demonstrating good performance in resolution improvement and seismic events continuity maintenance.

**Comment 2:** The authors must indicate why their study is of interest to the readers of the academic community

**Response 2:** This manuscript proposed a sequential convolutional neural network (SCNN) model to perform seismic high-resolution (HR) processing. There are some advantages: (1) the network architecture takes advantage of encoder-decoder network and residual learning, is appropriate for learning complex transformation between LR and HR trace pairs. (2) the adoption of 1D CNNs has advantage of fast processing speed than 2- or 3-D networks with considering the spatial information along depth, and is not limited by the lateral variations of the poststack or different prestack seismic data, suitable for processing both pre-stack and post-stack data; (3) the resolution of different wavelets is analyzed, and both well loggings and geological models are utilized to generate training dataset, considering some practical geological conditions. In summary, this manuscript contributes to CNN-based seismic HR processing deep learning and shows some advantages in fast and reliable HR processing for both poststack and prestack seismic data.
